# Unraveling the Mechanism of Platelet Aggregation Suppression by Monoterpenoids

**DOI:** 10.3390/bioengineering9010024

**Published:** 2022-01-10

**Authors:** Liliya E. Nikitina, Roman S. Pavelyev, Ilmir R. Gilfanov, Sergei V. Kiselev, Zulfiya R. Azizova, Alexander A. Ksenofontov, Pavel S. Bocharov, Elena V. Antina, Vladimir V. Klochkov, Ayzira F. Timerova, Ilfat Z. Rakhmatullin, Olga V. Ostolopovskaya, Mohammed A. Khelkhal, Sergei V. Boichuk, Aigul R. Galembikova, Natalia S. Andriutsa, Larisa L. Frolova, Alexander V. Kutchin, Airat R. Kayumov

**Affiliations:** 1 General and Organic Chemistry Department, Kazan State Medical University, 49 Butlerova st., 420012 Kazan, Russia; nikitl@mail.ru (L.E.N.); ilmir.gilfanov@gmail.com (I.R.G.); svkiselev08@mail.ru (S.V.K.); gzr27@yandex.ru (Z.R.A.); olga-ov.kirill@mail.ru (O.V.O.); boichuksergei@mail.ru (S.V.B.); ailuk000@mail.ru (A.R.G.); 2 Medical Physics Department, Kazan Federal University, 18 Kremlyovskaya st., 420008 Kazan, Russia; rpavelyev@gmail.com (R.S.P.); vladimir.klochkov@kpfu.ru (V.V.K.); aizirya96@bk.ru (A.F.T.); ilfat89rakhmatullin@gmail.com (I.Z.R.); amine.khelkhal@gmail.com (M.A.K.); kairatr@yandex.ru (A.R.K.); 3 G.A. Krestov Institute of Solution Chemistry of Russian Academy of Sciences, 1 Akademicheskaya st., 153045 Ivanovo, Russia; bochpavl@gmail.com (P.S.B.); eva@isc-ras.ru (E.V.A.); 4 Faculty of Fundamental and Applied Chemistry, Ivanovo State University of Chemistry and Technology, 7 Sheremetevskiy av., 153000 Ivanovo, Russia; 5 Institute of the Biological Design and Modelling of Complex Systems, I.M. Sechenov First Moscow State Medical University of the Ministry of Health of the Russian Federation, md. Edalgo 7, apt. 219, 108801 Moscow, Russia; natiandriutsa@mail.ru; 6 Institute of Chemistry, Federal Research Center, Komi Scientific Center, Ural Branch of the Russian Academy of Sciences, Pervomayskaya st. 48, 167982 Syktyvkar, Russia; frolova-ll@chemi.komisc.ru (L.L.F.); kutchin-av@chemi.komisc.ru (A.V.K.)

**Keywords:** S, N, O-containing monoterpenoids, platelet aggregation suppressing activity, 1D,2D solution-state NMR, cell membranes model, molecular docking, P2Y_12_ receptor

## Abstract

Platelet aggregation causes various diseases and therefore challenges the development of novel antiaggregatory drugs. In this study, we report the possible mechanism of platelet aggregation suppression by newly synthesized myrtenol-derived monoterpenoids carrying different heteroatoms (sulphur, oxygen, or nitrogen). Despite all tested compounds suppressed the platelet aggregation in vitro, the most significant effect was observed for the S-containing compounds. The molecular docking confirmed the putative interaction of all tested compounds with the platelet’s P2Y_12_ receptor suggesting that the anti-aggregation properties of monoterpenoids are implemented by blocking the P2Y_12_ function. The calculated binding force depended on heteroatom in monoterpenoids and significantly decreased with the exchanging of the sulphur atom with oxygen or nitrogen. On the other hand, in NMR studies on dodecyl phosphocholine (DPC) as a membrane model, only S-containing compound was found to be bound with DPC micelles surface. Meanwhile, no stable complexes between DPC micelles with either O- or N-containing compounds were observed. The binding of S-containing compound with cellular membrane reinforces the mechanical properties of the latter, thereby preventing its destabilization and subsequent clot formation on the phospholipid surface. Taken together, our data demonstrate that S-containing myrtenol-derived monoterpenoid suppresses the platelet aggregation in vitro via both membrane stabilization and blocking the P2Y_12_ receptor and, thus, appears as a promising agent for hemostasis control.

## 1. Introduction

The abnormal changes in blood coagulation leading to hemostasis disorders are the key elements of various obstetric and surgical pathology, cardiovascular, cerebrovascular, infectious, and immune diseases [1,2,3,4]. Nevertheless, the prevention and treatment of thrombosis and hemorrhagic conditions remain challenging and require a deeper insight into molecular mechanisms of blood coagulation and its regulation. It is worthwhile noting that most of the drugs suppressing the activity of platelets available to date do not guarantee effective prevention or treatment of thrombosis. Thus, the resistance of up to 61% of patients has been reported to aspirin, the most common and widely used anti-aggregation agent, which acts as an irreversible blocker of the cyclooxygenase enzymes and thromboxane A2 synthesis inhibitor [5]. The clopidogrel resistance, an inhibitor of well-known platelet receptors P2Y_12_, was reported to be in a range of 5–45% [6]. Therefore, the development of new agents for corrections of the hemostasis system disorders is strictly required.

Several studies have revealed that a large number of pathological processes generally don’t affect the hemostatic system directly, but influence the blood coagulation system by damaging the endothelium (for example, atherosclerosis, preeclampsia of pregnant women, etc.) [7]. Further, endothelial dysfunction activates platelet receptors and leads to platelet adhesion and aggregation under the influence of various inductors [8]. Thereby, developing novel substances suppressing the receptor activity of platelets seems to be a promising direction for investigation, while the role of the transformation of cell membranes in the activation of coagulation hemostasis is often overlooked.

Various agents interacting with cell membrane and thus modifying its properties seem promising agents for thrombosis control. Terpenes, a vast class of substances of membranotropic substances, provide membrane stabilization and decrease thrombogenic properties [9] via Van der Waals interactions with phospholipids of cell membranes. Moreover, sulphur-containing derivatives of monoterpenes have antifungal, anti-inflammatory, antibacterial, and anti-Helicobacter activity [10,11].

Previously we developed synthetic approaches for obtaining sulphur-containing monoterpenoids of different structures [12]. It has been found that among all the studied compounds, sodium ([(1R,2R,4R)-1,7,7-trimethylbicyclo[2.2.1]hept-2-yl] thio) acetate exhibits promising antiplatelet and anticoagulation activity and bioavailability because of its high solubility in water–alcohol solutions [13]. In addition, our study has reported that the distribution of the molecule within the cellular lipid membrane of platelets can directly influence the anticoagulant properties [13]. The NMR studies of this thioterpenoid interaction with phospholipid membrane revealed its membrane location with the shift to the membrane–water interface that apparently shields the hydrophobic interactions of coagulation factors with the membrane surface [13].

In this paper, we aim to demonstrate the crucial role of heteroatoms in platelet aggregation suppressing activity and anticoagulant properties of bicyclic monoterpenoids. For this reason, we synthesized three compounds based on (+) myrtenol with identical hydrophobic and hydrophilic fragments, differing only in heteroatoms (sulphur, oxygen, or nitrogen). The comparative study of their interaction with model phospholipid membranes by NMR spectroscopy and molecular docking with the platelet receptor P2Y_12_ allowed concluding that S-containing myrtenol-derived monoterpenoid suppresses the platelet aggregation in vitro via both membrane stabilization and blocking the P2Y_12_ receptor.

## 2. Materials and Methods

### 2.1. Chemistry

#### 2.1.1. Synthesis of 2-((((1S,5R)-6,6-Dimethylbicyclo[3.1.1]hept-2-en-2-yl)methyl)thio)ethan-1-ol (**4**)

The compound 2-mercaptoethanol (0.145 g, 1.86 mmol) was dissolved in a mixture of THF and DMF (70:30 V/V respectively, 20 mL), and K_2_CO_3_ (0.51 g, 3.69 mmol) was added. After 10 min, (+)-myrtenyl bromide **2** (0.4 g, 1.86 mmol) was added. The obtained mixture was stirred at room temperature for 24 h, and most of the solvent was removed under low pressure. The final product was purified with column chromatography (silica gel, hexane/ethyl acetate 4:1 V/V) to afford sulfide **4** (0.3 g, 76% yield) as a colorless oil. [α]^23^_D_  =  −5.0° (c 1; CH_3_OH). NMR ^1^H (CDCl_3_) δ, ppm: 0.83 (s, 3H), 1.13 (d, 1H, *J* = 8.7 Hz), 1.29 (s, 3H), 2.09 (br.s, 1H), 2.15–2.23 (m, 1H), 2.20–2.34 (q, 2H), 2.39–2.45 (m, 1H), 2.59–2.69 (m, 2H), 3.01 (d, 1H, *J* = 13.4 Hz), 3.11 (d, 1H, *J* = 13.4 Hz,), 3.65–3.74 (br.s., 2H), 5.37 (s, 1H). NMR ^13^C {^1^H} (CDCl_3_) δ, ppm: 21.26, 26.27, 31.43, 31.85, 34.16, 37.67, 38.27, 40.65, 45.18, 60.20, 120.49, 143.52. HRMS-ESI: m/z [M + H]^+^ calcd for C_12_H_21_OS: 213.1313; found: 213.1313.

#### 2.1.2. Synthesis of 2-((((1S,5R)-6,6-Dimethylbicyclo[3.1.1]hept-2-en-2-yl)methyl)amino)ethan-1-ol (**5**)

The compound 2-aminoethanol (0.227 g, 3.72 mmol) was dissolved in EtOH (20 mL), and then (+)-myrtenyl bromide **2** (0.2 g, 0.93 mmol) was added. The obtained mixture was stirred at 50 °C for 24 h, and most of the solvent was removed under low pressure. The final product was purified with column chromatography (silica gel, acetone) to afford amine **5** (0.086 g, 47% yield) as amorphous, yellow crystals. [α]^23^_D_  =  24.4° (c 0.86; CHCl_3_). NMR ^1^H (CDCl_3_) δ, ppm: 0.85 (s, 3H), 1.16 (d, 1H, *J* = 8.7 Hz), 1.31 (s, 3H), 2.11 (br.s, 2H), 2.22–2.34 (q, 2H), 2.39–2.44 (m, 1H), 2.74–2.78 (m, 2H), 3.15 (br.q, 2H, *J* = 1.8 Hz), 3.66 (t, 2H, *J* = 5.3 Hz), 5.4 (m, 1H). NMR ^13^C {^1^H} (CDCl_3_) δ, ppm: 21.29, 26.08, 31.66, 31.82, 38.34, 40.47, 44.20, 49.20, 52.74, 57.95, 126.11, 139.82. HRMS-ESI: m/z [M + H]^+^ calcd for C_12_H_22_NO: 196.1701; found: 196.1701.

### 2.2. Platelet Aggregation Assays

The blood aggregation assays were performed as described in [13]. The venous blood was obtained by cubital vein puncture from healthy volunteers (average 30 ± 6-year-old) taking no drugs affecting clotting factors or platelet function for at least 7–10 days before. All participants gave informed consent from all subjects and approval by the Ethical Committee of Kazan State Medical University. All procedures were carried out following the approved guidelines. All subjects have been provided with the written informed consent following the Declaration of Helsinki. The blood was stabilized with a 3.8% sodium citrate solution and pooled. Then, for the platelet-rich plasma preparation, the blood was centrifuged (10 min, 1000 rpm), the upper layer of plasma was moved into another tube and the remaining portion of the blood was centrifuged (20 min, 3000 rpm) to obtain platelet-poor plasma (further used for dilution of platelet-rich plasma if required). The aggregating activity of platelets was determined by a “Chrono-Log Corporation” analyzer (Havertown, PA, USA) according to the Born approach (1962). Briefly, 0.05 mL of compound (20 mM solution in the water solution of ethyl alcohol) was added to 0.45 mL of plasma and incubated for 5 min at 37 °C. The final concentration in human blood plasma of all compounds was 2 mM. As a control, the solvent (3% water solution of ethyl alcohol) was added to the plasma. The platelet aggregation was started by adding ADP (5 μM), adrenaline (10 μM), collagen (2 μg/mL), arachidonic acid (0.5 mM), and ristocetin (1 mg/mL).

### 2.3. Molecular Docking

To define the most probable binding site of compounds **3–5** and ticagrelor active metabolite (AM) with P2Y_12_, a “blind” docking by using AutoDock 4.2 [14] was performed. The structure of the human P2Y_12_ receptor was taken from the Protein Data Bank (No. 4NTJ). The compounds **3–5** and ticagrelor AM structures were fully optimized by CAMB3LYP/def2-TZVP [15,16] in PC GAMESS v.12 [17]. In AutoDock, the grid box 90 Å × 126 Å × 120 Å with step in 0.7 Å was chosen to study the possibility of ligands’ localization within the protein. For each calculation, 50 separate runs were made using Lamarck’s genetic algorithm, which were stopped after a maximum of 25 million energy calculations. Ligand-receptor complexes were sorted by root-mean-square deviation. For each complex, the conformation with the lowest energy was chosen as the most stable. Molecular plots were made via UCSF Chimera [18].

To refine the binding sites and energy parameters, specific docking was performed in the GOLD v. 2021 software. All atoms within a radius of 20 Å from the ligand were selected. For all ligands, 50 separate runs were performed as in the case of blind docking. GoldScore with rescoring by ChemScore was chosen as an approach to evaluate the binding efficiency. In the GA settings, the highest search efficiency of 200% was chosen to get the most accurate results [19].

The algorithm selected for molecular docking was tested on the basis of docking «P2Y_12_ (PDB: 4NTJ)-AZD1283» [20], «P2Y_12_ (PDB: 4PXZ)-2MeSATP» [21], and «P2Y_12_ (PDB: 4PY0)-2MeSADP» [21] systems. The test results showed that chosen docking algorithm reproduces well the experimentally obtained (X-Ray) structural features of the P2Y_12_ binding sites with antagonists.

### 2.4. NMR Experiments

Solution state NMR experiments were carried out on the Bruker AVANCE III HD NMR spectrometer operating at 700 MHz (^1^H) frequency equipped with a 5 mm probe, employing standard Bruker TOPSPIN-NMR software at T = 300 K. ^1^H NMR spectra were recorded using 90° pulses with a duration of 7.0 μs delay between pulses of 2 s, a spectrum width of 12 ppm and a minimum of sixteen scans. Complete assignment of the ^1^H NMR spectrum of the compound was accomplished by 2D ^1^H-^1^H COSY, ^1^H-^13^C HSQC, and ^1^H-^13^C HMBC NMR experiments. Chemical shifts were given in values of ppm, referenced to a residual solvent signal (^1^H in D_2_O—4.78 ppm; ^1^H in CDCl_3_—7.26 ppm). The samples were prepared by dissolving in D_2_O with concentrations of 5.09, 4.71, and 5.12 mM respectively. The solution volume was 0.6 mL. Micelles of dodecyl phosphocholine (DPC) were obtained by dissolving DPC in D_2_O to a final concentration of 45.4 mM. 2D NOESY experiments were performed with phase-sensitive techniques with presaturation. The relaxation delay was set to 2 s. The mixing time value in the 2D NOESY experiment was 0.3 s. 2D DOSY experiments were carried out through the pulse sequence with bipolar gradients pulse longitudinal eddy current delay (LEDBPGP2S). The pulse gradients length was enhanced for each diffusion delay in an attempt to obtain a 2% residual signal at 95% of the maximum gradient strength.

### 2.5. Cells Viability MTS Assay

BJ tert fibroblasts were seeded in 96-well flat-bottomed plates (Corning Inc., Corning, NY, USA) and cultured for 24 h. The cells were further treated with tested compounds at concentrations as indicated or DMSO as a control for 48–72 h. To assess the viability of cells, the MTS reagent (Promega, Madison, WI, USA) was added to the culture in concentrations as recommended by the manufacturer, and incubation was followed for 1 h. The MTS reduction product was measured using a MultiScan FC plate reader (Thermo Fisher Scientific, Waltham, MA, USA) at 492 nm. IC50 was calculated as the concentration of the compound inhibiting the cell growth by 50% after 48–72 h. Data were normalized to the DMSO-treated (control) group.

### 2.6. Statistic Analysis

All procedures were performed by using Graph Pad Prism 6. The results were analyzed with the Kolmogorov–Smirnov test and the Kruskal–Wallis test.

## 3. Results

### 3.1. Synthesis of Monoterpenoids and Their Effect on Platelet aggregation

**Scheme 1:**
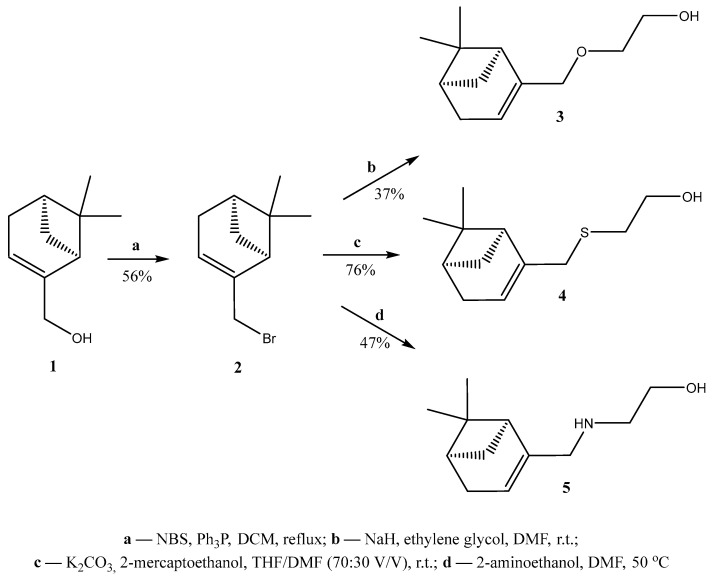
Synthesis of compounds **2–5**.

Compounds **2–5** were synthesized based on (+)—myrtenol **1**, which was prepared by the oxidation of (+) α-pinene with tert-butyl hydroperoxide in the presence of catalytic amounts of SeO_2_ according to a well-known procedure [22]. The synthesis and the physical and chemical properties of bromide **2** and ether **3** were described earlier in [23,24,25] and all the characteristics of compound **3** were consistent with previously published data. Sulfide **4** and amine **5** were synthesized for the first time (Scheme 1).

Firstly, myrtenyl bromide **2** was synthesized by using N-bromosuccinimide in the presence of triphenylphosphine in methylene chloride. The reaction was carried out for four hours at high temperature and the product yield after purification by column chromatography on silica gel with hexane was 56%. Further, ether **3** was prepared from bromide **2** using ethylene glycol and sodium hydride in DMF and the product yield was 37%. Next, sulfide **4** was synthesized in the presence of potash from bromide **2** and mercaptoethanol in a THF/DMF mixture with a ratio of 70:30 (*v*/*v*) at room temperature and the product yield reached 76%. Finally, amine **5** was obtained by alkylation of the monoethanolamine nitrogen atom with myrtenyl bromide **2** in DMF at room temperature with a yield of 47%. The synthesis of all compounds is described in detail in Materials and Methods section.

**Figure 1:**
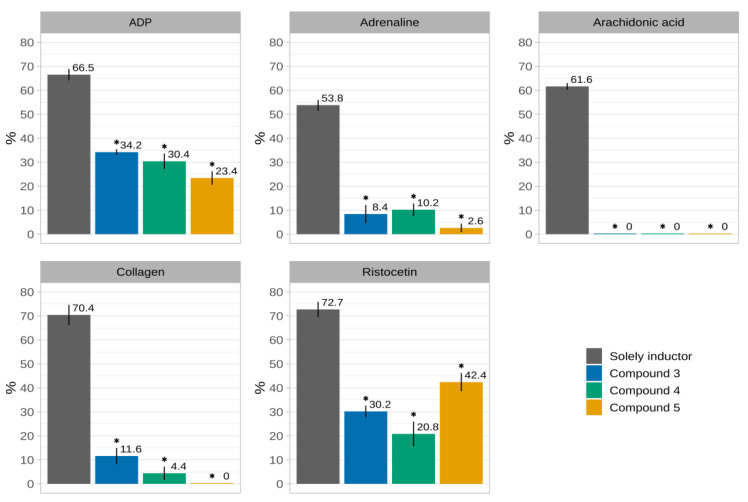
Influence of compounds **3**–**5** on platelets’ activation induced in vitro. * *p* ˂ 0.001 in comparison with indicators without substance.

Platelet aggregation was induced by their activation by various physiological or pathological factors (ADP, adrenaline, arachidonic acid, collagen, ristocetin) as suggested previously [8]. Compounds **3–5** significantly suppressed the platelet aggregation induced by ADP, collagen, adrenaline, and ristocetin and completely abrogated platelet aggregation induced by arachidonic acid (Figure 1, Table 1).

Arachidonic acid activates platelets directly by penetrating the cell membrane in contrast to other inducers acting via receptors and reduces cyclic adenine monophosphate (cAMP) concentration. Therefore, the loss of platelet aggregation in the presence of compounds **3**–**5** could be attributed to the changes in the permeability of platelet membranes, which in turn could be associated with additional intermolecular interactions between hydrophobic parts of the terpenes and the phospholipids of the membrane. As well, the decrease of aggregation in the presence of terpenoids **3–5** seems to be associated with increased intracellular cAMP formation due to membrane lipids peroxidation blocking. In addition, integration of the hydrophobic part of compounds **3–5** into the cellular membrane induces its stabilization and thereby prevents the utilization of the phosphatidylcholine molecules from the external layer as a formation source of lipid peroxidation products, which in turn will trigger the platelet aggregation mechanisms.

### 3.2. Molecular Docking

The P2Y receptors are G-protein-coupled receptors (GPCRs) for extracellular adenine and uracil nucleotides. Human platelets express P2Y_1_ and P2Y_12_ receptors, which bind adenosine diphosphate (ADP), the first known low molecular weight platelet agonist. Activation of both P2Y receptors is necessary for normal platelet responses to ADP [26], whereas the P2Y_12_ receptor increases the sensitivity of platelets to agonists. Since the aggregation induction by ADP and other effectors was significantly suppressed in presence of monoterpenoids, we suggested their interaction with the P2Y_12_ receptor.

**Figure 2:**
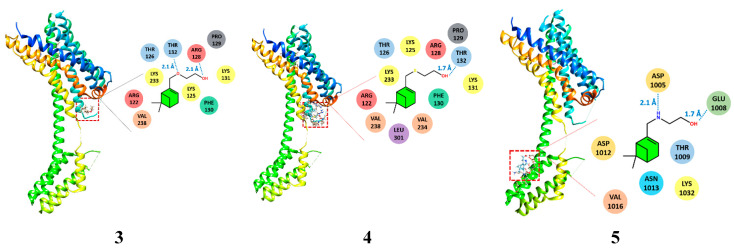
The molecular docking model of interaction of compounds **3–5** with P2Y_12_. The blue, dashed lines show the formation and distance of the H-bonds.

The interaction of compounds **3–5** with the P2Y_12_ receptor (responsible for the platelet aggregation process) was assessed by using the molecular docking approach. Blind molecular docking results revealed that all compounds are located in the intracellular region of the P2Y_12_ receptor closer to the C-terminal region of the receptor (Figure 1). All studied compounds interact with P2Y_12_ via the hydrogen bonds formation by ether and hydroxyl groups of ether **3**, the hydroxyl group of sulfide **4**, and the amino and hydroxyl groups of amine **5**. These groups are located close (distance from 1.7 Å to 2.1 Å) to side-chain groups of amino acid residues (ARG128, THR132, GLU1004, ASP1005, GLU1008) forming binding sites in the P2Y_12_ receptor (Figure 2, Table 2).

**Figure 3:**
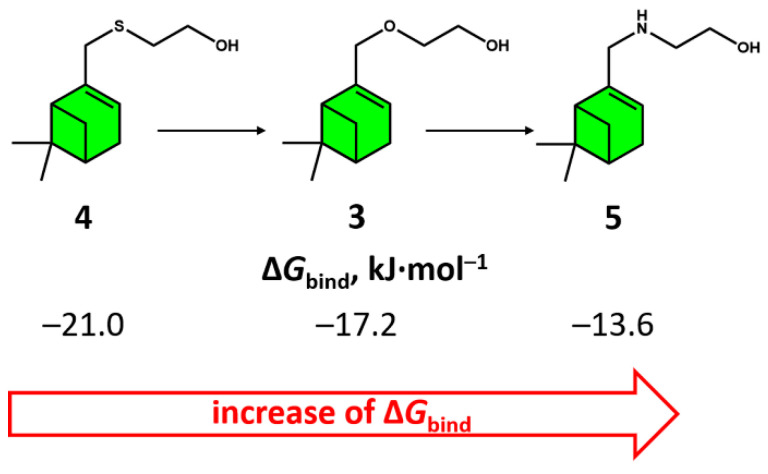
The change of the Gibbs free energy (Δ*G*_bind_) of terpenoids **3–5** binding to P2Y_12_.

To study the Gibbs free energy of terpenoids binding to P2Y_12,_ at the next stage the studied compounds were redocked by using the GOLD software tool. Interestingly, the Δ*G*_bind_ increased in series of heteroatom changes from sulphur to oxygen and nitrogen (Figure 3), suggesting the most stable complex of P2Y_12_ with sulfide **4**.

These data allow assuming that compound **5** suppresses the platelet aggregation rather via membrane stabilization than over the receptor blockage. The hydrophobic part is incorporated into the phospholipid membrane, while the hydrophilic tail is weakly retained by the intracellular region of P2Y_12_ receptor.

**Figure 4:**
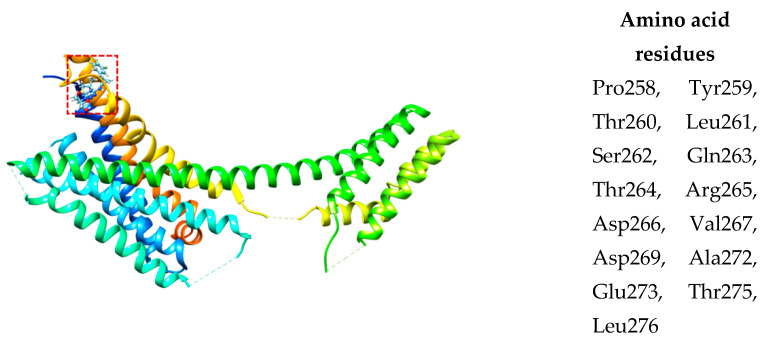

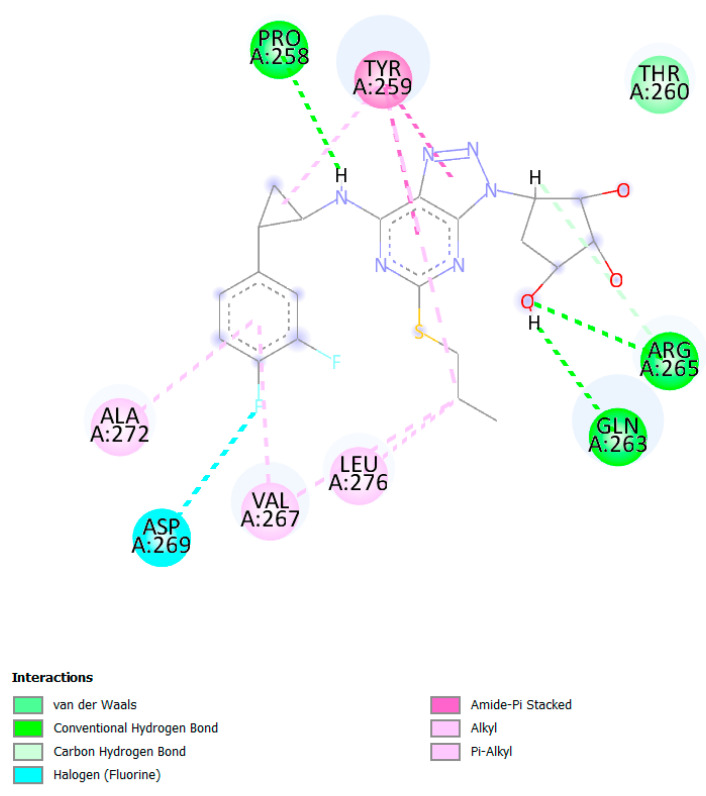
The molecular docking model of ticagrelor AM in the binding site of P2Y_12_.

To evaluate the potential use of compound **4** as a platelet aggregation suppressor, we compared its affinity to P2Y_12_ with that of ticagrelor AM, a commercially available agent preventing the platelet aggregation. Molecular docking of the ticagrelor AM–P2Y_12_ interaction demonstrated that ticagrelor AM is localized near the extracellular N-terminal region of the P2Y_12_ receptor (Figure 4). The Ticagrelor AM has a significantly higher affinity to the P2Y_12_ receptor (binding energy-40.1 kJ.mol^−1^) and the binding site does not fit with those of compounds **3–5**. This suggests that the mechanism of P2Y_12_ receptor blocking by **4** differs for Ticagrelor AM and compounds **3–5**. By contrast, several researchers reported series of P2Y_12_ inhibitors binding with various sites on the receptor molecule assuming different possible pathways of P2Y_12_ receptor blocking.

### 3.3. NMR Experiments

NMR spectroscopy was used to evaluate the interaction between compounds **3–5** and the model cell membrane. The interaction of monoterpenoids with the cell membrane has already been investigated in our previous studies [27,28,29]. Here, the dodecyl phosphocholine (DPC) micelles solution in water were used as model systems mimicking the surface of the cell membrane. DPC has the same zwitterionic head group as phosphatidylcholine and can be used as a simple model for eukaryotic membranes. At the same time, DPC micelles are much smaller than phospholipid bilayers and, therefore, more suitable for NMR spectroscopy in solution [30,31,32,33,34,35].

**Figure 5:**
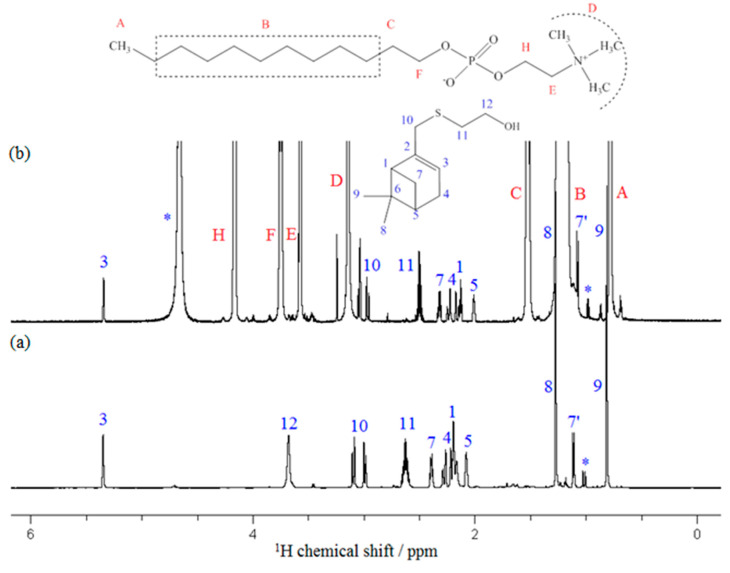
The ^1^H NMR spectra of compound **4** in CDCl_3_ (**a**) and D_2_O+DPC (**b**) solutions at 300 K. Numbers designate protons of compound **4**, letters A–H corresponds to DPC protons. The chemical structures of the DPC molecule and compound **4** are shown on the top of the figure. The solvent and impurity signals are marked by asterisks (*).

^1^H NMR spectrum of compound **4** has been significantly changed after the addition of DPC micelles (Figure 5). The signals of CH_2_-10 and CH_2_-11 protons have been narrowed. Unfortunately, line shape changes of the signals CH_3_-8, 9, and CH_2_-12 could not be analyzed due to overlap with DPC signals. However, shifts to the lower field of H-1, H-4, H-5, H-7 allowed the observation of signals. These differences between the ^1^H NMR spectra of the compound in pure CDCl_3_ solvent and the D_2_O + DPC micelles system are caused by the interaction of the studied monoterpenoid with the membrane model.

**Figure 6:**
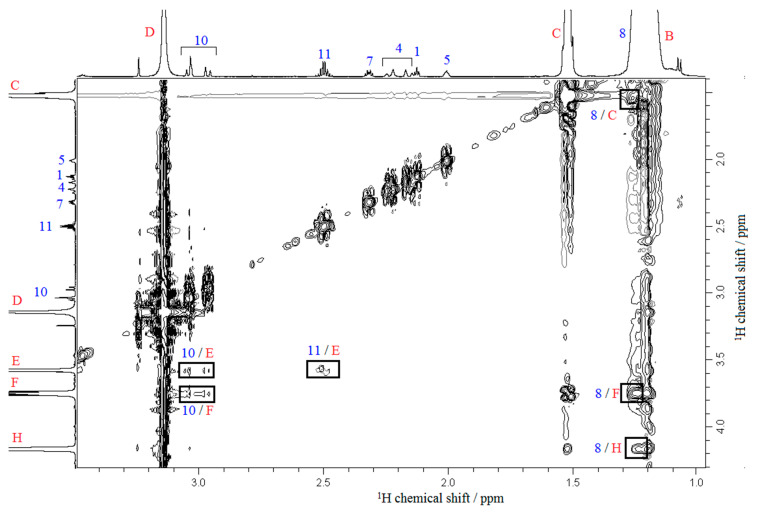
The fragment of 2D NOESY NMR spectrum of compound **4** in DPC solution at 300 K. Numbers designate protons of compound **4**, letters B–H belong to DPC protons. Intermolecular cross-peaks are highlighted by bold black frames.

To clarify the mechanism of the complex formation between compound **4** and DPC micelle, 2D NOESY NMR experiments were carried out (Figure 6). Several non-trivial intermolecular nuclear Overhauser effects (NOE) indicating the close spatial location of the corresponding chemical groups of the studied compound and DPC micelle were observed. The cross-peaks between the signals C, E, F, and H of DPC and the signals CH_3_-8, CH_2_-10, and CH_2_-11 of compound **4** serve as evidence of interaction of the studied compound with the surface of DPC micelle.

**Figure 7:**
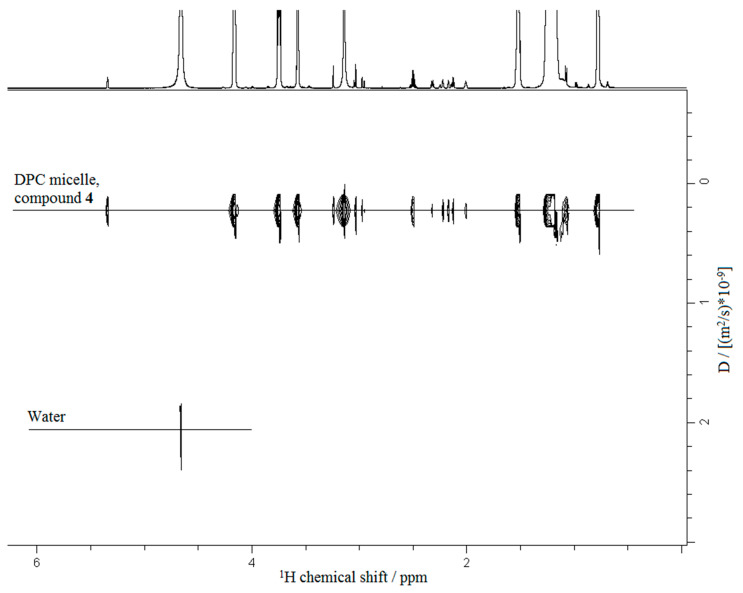
The 2D DOSY NMR spectrum of compound **4** in DPC/D_2_O solution at temperature 300 K.

So that we could check whether compound **4** intercalates into the membrane mimetic, 2D DOSY NMR experiments were carried out (Figure 7). Insignificantly different diffusion coefficients were observed for protons of DPC molecule (D = (9.281 ± 0.742) × 10^−11^ m^2^/s) and for compound **4** (D = (9.798 ± 0.784) × 10^−11^ m^2^/s) indicating that compound **4** binds to DPC micelles surface. Thus, solution-state NMR data confirm the stable complex formation between DPC micelles and the studied compound **4**.

To check if compounds **3** and **5** interact with the membrane model, 2D DOSY experiments were also carried out. Significantly different diffusion coefficients were observed for protons of DPC molecule (D = (9.566 ± 0.765) ×10^−11^ m^2^/s) and for compound **3** (D = (13.488 ± 1.079) × 10^−11^ m^2^/s). Furthermore, the 2D DOSY NMR experiment showed that compound **5** and DPC micelles had rather different self-diffusion coefficients in D_2_O solution ((16.360 ± 1.309) × 10^−11^ and (9.560 ± 0.765) × 10^−11^ m^2^/s, respectively) suggesting that compounds **3** and **5** do not bind to DPC micelles.

### 3.4. Cytotoxicity

**Figure 8:**
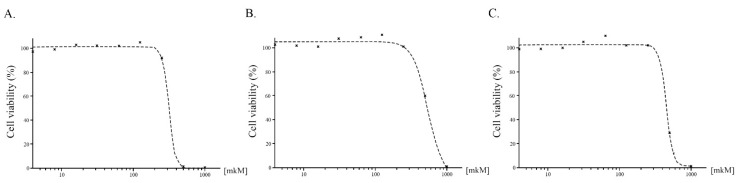
The viability of BJ tert human fibroblasts treated compound **3**–**5** (**A**–**C**, respectively) for 72 h. The data was normalized to solvent-treated controls. Values are the means ± standard deviation (n = 3). Statistical analyses (Student’s *t*-test) were performed using Statistical software program version 7.0 (S.A. Glantz, McGraw Hill Education, NY, USA). *p* < 0.05 was considered to indicate a statistically significant difference. Half-inhibitory concentration (IC50) was determined by using the IC50 Tool Kit (http://ic50.tk/, accessed on 15 February 2021). The IC50 value is graphically represented as a proper sigmoid curve (**A**–**C**).

The cytotoxic properties of the compounds **3–5** were evaluated in metabolic MTS assay. For that, BJ tert human fibroblasts (ATCC, Manassas, VA, USA) were seeded in 96-well culture plates (Corning Inc. Corning, NY, USA) and allowed to attach and grow for 24 h before treatment. Then, compounds **3–5** were added and cultivation was followed for 72h and the viability of cells has been evaluated. Compounds **3–5** exhibited the cytotoxic properties against BJ tert fibroblasts at concentrations higher than 300 µM (Figure 8A–C), with IC50 values lying in the range of 300–600 µM (Table 3).

## 4. Conclusions

Taken together, all studied monoterpenoid compounds suppressed the platelet aggregation in vitro apparently via blocking their P2Y_12_ receptor activity. Molecular docking indicated that the binding force with platelet P2Y_12_ receptor depends on heteroatom in monoterpenoids and decreases in series from sulphur to oxygen and nitrogen atoms. Detailed NMR studies using dodecyl phosphocholine (DPC) as membrane model revealed that only S-containing compound binds to DPC micelles surface. This binding reinforces the mechanical properties of the cell membranes and prevents destabilizing and following clot formation on the phospholipid surface. No confirmation of stable complex formation between DPC micelles and O- and N-containing compounds were obtained from solution-state NMR data. Most likely, for all the studied compounds, both mechanisms of action are realized—receptor and membrane factors, but in the case of the S-containing compound, both factors are more pronounced. On a wider lever, considering the low toxicity, the ability to block induced-aggregation, S-containing monoterpenoids seem to be promising agents for the blood products stabilization, treatment, and prevention of thrombophilia.

## Figures and Tables

**Table 1:** Influence of compounds **3**–**5** on platelets activation induced in vitro. The percent of induction is shown.

Inductor	Solely Inductor	3	4	5
ADP	66.5 ± 2.4	34.2 ± 1.2 *	30.4 ± 3.2 *	23.4 ± 2.8 *
adrenaline	53.8 ± 2.2	8.4 ± 3.8 *	10.2 ± 2.6 *	2.6 ± 1.8 *
arachidonic acid	61.6 ± 1.4	0 *	0 *	0 *
collagen	70.4 ± 4.2	11.6 ± 3.4 *	4.4 ± 2.8 *	0 *
ristocetin	72.7 ± 3.2	30.2 ± 2.4 *	20.8 ± 5.2 *	42.4 ± 3.8 *

* *p* ˂ 0.001 in comparison with indicators without substance.

**Table 2:** Amino acid residues of P2Y_12_ binding sites.

Compound	Amino Acid Residues
**3**	ARG122, LYS125, THR126, ARG128, PRO129, PHE130, LYS131, THR132, LYS232, LYS233, VAL238
**4**	ARG122, LYS125, THR126, ARG128, PRO129, PHE130, LYS131, THR132, LYS233, VAL234, VAL238, LEU301
**5**	ASP1005, GLU1008, THR1009, ASP1012, ASN1013, VAL1016, LYS1032

**Table 3:** IC_50_ values for compounds **3–5** on BJ tert human fibroblasts.

Compound	IC_50_ Value, µM
**3**	548 ± 52.7
**4**	314 ± 46.5
**5**	450 ± 71.6

## Data Availability

Data are available by request to corresponding author.
